# HLA and KIR genetic association and NK cells in anti-NMDAR encephalitis

**DOI:** 10.3389/fimmu.2024.1423149

**Published:** 2024-07-10

**Authors:** Vicente Peris Sempere, Guo Luo, Sergio Muñiz-Castrillo, Anne-Laurie Pinto, Géraldine Picard, Véronique Rogemond, Maarten J. Titulaer, Carsten Finke, Frank Leypoldt, Gregor Kuhlenbäumer, GENERATE study group, Hannah F. Jones, Russell C. Dale, Sophie Binks, Sarosh R. Irani, Anna E. Bastiaansen, Juna M. de Vries, Marienke A. A. M. de Bruijn, Dave L. Roelen, Tae-Joon Kim, Kon Chu, Soon-Tae Lee, Takamichi Kanbayashi, Nicholas R. Pollock, Katherine M. Kichula, Abigail Mumme-Monheit, Jérôme Honnorat, Paul J. Norman, Emmanuel Mignot

**Affiliations:** ^1^ Stanford Center for Sleep Science and Medicine, Stanford University, Palo Alto, CA, United States; ^2^ French Reference Center on Paraneoplastic Neurological Syndrome and Autoimmune Encephalitis, Hospices Civils de Lyon, Lyon, France; ^3^ Institut MeLiS INSERM U1314/CNRS UMR 5284, Université Claude Bernard Lyon 1, Lyon, France; ^4^ Department of Neurology, Erasmus Medical Center, Rotterdam, Netherlands; ^5^ Department of Neurology and Experimental Neurology, Charité-Universitätsmedizin Berlin, Berlin, Germany; ^6^ Berlin School of Mind and Brain, Humboldt-Universität zu Berlin, Berlin, Germany; ^7^ Department of Neurology, Christian-Albrechts-University/University Hospital Schleswig-Holstein, Kiel, Germany; ^8^ Neuroimmunology, Institute of Clinical Chemistry, University Hospital Schleswig-Holstein Kiel/Lübeck, Kiel, Germany; ^9^ German Network for Research on Autoimmune Encephalitis, Germany; ^10^ Starship Hospital, Centre for Brain Research, Faculty of Medical and Health Sciences, University of Auckland, Auckland, New Zealand; ^11^ Kids Neuroscience Centre, Children’s Hospital at Westmead clinical school, University of Sydney, Sydney, NSW, Australia; ^12^ Oxford Autoimmune Neurology Group, Nuffield Department of Clinical Neurosciences, University of Oxford, Oxford, United Kingdom; ^13^ Department of Neurology, John Radcliffe Hospital, Oxford, United Kingdom; ^14^ Departments of Neurology and Neurosciences, Mayo Clinic, Jacksonville, FL, United States; ^15^ Department of Immunogenetics and Transplantation Immunology, Leiden University Medical Center, Leiden, Netherlands; ^16^ Department of Neurology, Ajou University School of Medicine, Suwon, Republic of Korea; ^17^ Department of Neurology, Seoul National University Hospital, Seoul National University College of Medicine, Seoul, Republic of Korea; ^18^ Department of Neurology, Teikyo University School of Medicine, Tokyo, Japan; ^19^ Department of Biomedical Informatics, University of Colorado School of Medicine, Aurora, CO, United States; ^20^ Department of Immunology and Microbiology, University of Colorado School of Medicine, Aurora, CO, United States

**Keywords:** anti-NMDAR encephalitis, KIR, KIR3DL3, KIR2DL4, HLA, DQA1, DQB1, DRB1

## Abstract

**Introduction:**

Genetic predisposition to autoimmune encephalitis with antibodies against N-methyl-D-aspartate receptor (NMDAR) is poorly understood. Given the diversity of associated environmental factors (tumors, infections), we hypothesized that human leukocyte antigen (*HLA*) and killer-cell immunoglobulin-like receptors (*KIR*), two extremely polymorphic gene complexes key to the immune system, might be relevant for the genetic predisposition to anti-NMDAR encephalitis. Notably, KIR are chiefly expressed by Natural Killer (NK) cells, recognize distinct HLA class I allotypes and play a major role in anti-tumor and anti-infection responses.

**Methods:**

We conducted a Genome Wide Association Study (GWAS) with subsequent control-matching using Principal Component Analysis (PCA) and *HLA* imputation, in a multi-ethnic cohort of anti-NMDAR encephalitis (n=479); *KIR* and *HLA* were further sequenced in a large subsample (n=323). PCA-controlled logistic regression was then conducted for carrier frequencies (*HLA* and *KIR*) and copy number variation (*KIR*). HLA-KIR interaction associations were also modeled. Additionally, single cell sequencing was conducted in peripheral blood mononuclear cells from 16 cases and 16 controls, NK cells were sorted and phenotyped.

**Results:**

Anti-NMDAR encephalitis showed a weak *HLA* association with DRB1*01:01~DQA1*01:01~DQB1*05:01 (OR=1.57, 1.51, 1.45; respectively), and DRB1*11:01 (OR=1.60); these effects were stronger in European descendants and in patients without an underlying ovarian teratoma. More interestingly, we found increased copy number variation of *KIR2DL5B* (OR=1.72), principally due to an overrepresentation of KIR2DL5B*00201. Further, we identified two allele associations in framework genes, KIR2DL4*00103 (25.4% vs. 12.5% in controls, OR=1.98) and KIR3DL3*00302 (5.3% vs. 1.3%, OR=4.44). Notably, the ligands of these KIR2DL4 and KIR3DL3, respectively, HLA-G and HHLA2, are known to act as immune checkpoint with immunosuppressive functions. However, we did not find differences in specific KIR-HLA ligand interactions or HLA-G polymorphisms between cases and controls. Similarly, gene expression of CD56^dim^ or CD56^bright^ NK cells did not differ between cases and controls.

**Discussion:**

Our observations for the first time suggest that the HLA-KIR axis might be involved in anti-NMDAR encephalitis. While the genetic risk conferred by the identified polymorphisms appears small, a role of this axis in the pathophysiology of this disease appears highly plausible and should be analyzed in future studies.

## Introduction

Encephalitis with antibodies against N-methyl-D-aspartate receptor (NMDAR) is one of the most common autoimmune encephalitides and has recently been considered of intermediate risk for a paraneoplastic origin based on a tumor association rate of approximately 40% (1, 2). However, tumors are extremely rare in children and young men, ovarian teratomas are present in about 50% of women during their fertile age and heterogeneous carcinomas identified in 30% of elderly patients (3–6). Strikingly, it has been proven that ovarian teratomas associated with anti-NMDAR encephalitis harbor particular immunopathological characteristics, including more frequent glial GluN1 expression, and harbor B cells whose B cell receptors directly bind the NMDAR (7–9). Similarly, other malignant tumors may also express this NMDAR subunit (4). Additionally, there are rare cases of autoimmune encephalitis with NMDAR antibodies following herpetic encephalitis (10). However, despite 70% of patients presenting with prodromal, mild viral-like symptoms, the pathogenesis of the remaining cases of anti-NMDAR encephalitis remains obscure (11). Thus, although the aforementioned tumors and perhaps herpetic encephalitis seem to be able to trigger an autoimmune reaction in a subset of subjects, the mechanisms underlying the initiation of the autoimmune response are still unknown in most cases. A genetic predisposition conferring some risk has been postulated but results remain heavily debated (12–16).

The human leukocyte antigen (*HLA*) region is one of the most gene dense, complex, and polymorphic regions of the human genome. It harbors polymorphic genes involved in antigen presentation, where the HLA subtypes modulate immune responses to specific peptides and antigens. As a result, genetic associations with *HLA* are commonly found in infectious diseases, although these are typically strongest with autoimmune disorders, including specific subtypes of autoimmune encephalitis (12, 17–22). To date, weak *HLA* association findings have been reported in small samples of patients with anti-NMDAR encephalitis (13, 14), although these were not replicated in a large, recent cohort (16). Even more recently, however, a genome-wide association study (GWAS) performed in a large Chinese cohort described strong associations with DQB1*05:02, A*11:01 and A*02:07 (23).

Interestingly, some HLA class I molecules are also ligands for killer-cell immunoglobulin-like receptors (KIRs), a less studied group of polymorphic immune regulators. KIRs are immune surface receptors encoded by up to 13 polymorphic genes in each individual (24, 25). KIRs are principally expressed by natural killer (NK) cells, which play roles in immune responses against both cancer and infections. Recent data also suggest specific KIRs also regulate tolerance of CD8^+^ T cells (26). KIRs are key to anti-tumor and anti-viral immune responses (27, 28), and deficiencies in NK cell numbers are associated with increased susceptibility to infection by Herpesviridae, such as human cytomegalovirus (CMV), Epstein-Barr virus (EBV), herpes simplex virus (HSV) and varicella-zoster virus, as well as by human papillomavirus and other viruses (29). Because of the above, NK cells and associated receptors are plausible candidates genetically predisposing to anti-NMDAR encephalitis. Herein, we aimed to investigate potential *HLA* and *KIR* associations in a large, multiethnic cohort of anti-NMDAR encephalitis.

The analysis of *KIR* associations is complex, as specific receptors may become functional only in the presence of their cognate HLA ligand. The ligands include subsets of HLA-A, B and C alleles that carry A3/11, Bw4, C1 or C2 motifs (24, 25, 27, 30, 31). Thus, a given individual may not carry all the KIR or their HLA class I ligands. To explore a possible *HLA-KIR* association, we first assume that the *KIR* allele association could be strong enough to manifest dominantly independent of the presence of its ligand in the same individual. This was followed by an analysis of the number of existing paired interactions in each patient versus controls, as previously done in other studies (32–34). Doing so, we also explored both presence and number of *KIR* genes that are copy number variations in cases versus controls, allelic differences within these genes when present in cases versus controls, and presence or absence of *KIR-HLA* ligand pairs in cases versus controls. Finally, as these receptors can be either inhibitory or activating, we approximated and compared the amount of inhibitory and activating inputs NK cells were likely to receive in cases versus controls.

## Materials and methods

### Patients and controls

A multi-ethnic cohort of patients fulfilling diagnostic criteria for anti-NMDAR encephalitis (35) and available DNA were retrospectively recruited (**Supplementary Table 1**). This sample contains GWAS typed samples of 479 cases and 2,806 PCA (principal component analysis) and genotyping platform-matched controls (**Supplementary Table 1**). A total of 15 patients with post-herpetic autoimmune encephalitis and presence of NMDAR antibodies were excluded from this study, given their known clinical, immunological and genetic particularities (10, 36). All samples were de-identified. This cohort was primarily used for *HLA* imputation and allele comparison across the entire cohort.

A subsample of 323 cases and 1,519 controls, the main object of this study, was drawn from this larger sample of anti-NMDAR encephalitis cases and was analyzed using *KIR* and *HLA* sequencing. Ethnicity distribution for the sequenced sample is reported in **Supplementary Table 2**. In this sample, 71 (21.9%) cases had teratomas, 12 (3.7%) had other tumors and 238 (73.7%) were non-paraneoplastic; information regarding a possible underlying tumor was unavailable for 2 (0.6%) patients.

### Genome wide association

Patients and controls were genotyped using Affymetrix or Illumina chips (see **Supplementary Table 1**). Only high-imputation calls (R2>0.9) were used to conduct genotype data calculations. All genotype data operations were performed using PLINK 1.9 (www.cog-genomics.org/plink/1.9/) (37). Patients were genome-wide imputed to the 1000 Genome Phase III dataset (38) after haplotype phasing and merged using QCTOOL and were subsequently PCA-matched by ethnicity to their closest 10 controls based on Euclidean distance using PLINK. Ethnicity was manually defined from PCAs. Genotypes were used, together with geographic origin, to identify PCA matched controls for *KIR/HLA* sequencing, and to conduct *HLA* imputation.

### *KIR* and *HLA* sequencing

The subset of 323 cases and 1,519 PCA-matched control samples were sequenced for all *KIR* and *HLA* genes, as previously described (39–41). After sequencing, raw FASTQ files were analyzed using our custom bioinformatics pipeline PING (Pushing Immunogenetics into the Next Generation) to obtain *KIR* gene content and *KIR* allelic genotypes from next-generation sequencing (NGS) data (39). We applied an updated version of the pipeline that precisely determines the copy number of each locus through multiple alignment and filtration steps, also accurately identifying *KIR* genotypes. The updated pipeline increased the accuracy of *KIR* genotype determination and is publicly available (40, 42). In cases where *KIR* sequencing was ambiguous due to multiple allele combinations, the most frequent set of alleles was selected based on previously reported *KIR* frequencies (41). In parallel with *KIR*, *HLA class I* and *class II* alleles were determined from the sequence data using the consensus calls obtained using three algorithms: NGSengine^®^ 2.10.0 (GenDX, Utrecht, the Netherlands), HLA Explore™ (Omixon Biocomputing Ltd. Budapest, Hungary) and HLA*LA (43), as previously described (44). Included were genotyping for alleles of *HLA-G*, and *HLA-G* 14-bp insertion/deletion genotyping, a 3’UTR variant suggested to modulate expression (45).

### *HLA* imputation

*HLA* imputation was also conducted in a larger cohort of 479 cases and 2,806 PCA-matched controls using HLA Genotype Imputation with Attribute Bagging (HIBAG), performed post-genome wide-imputation and using ethnic and platform-specific models (**Supplementary Table 1**) (21, 46). *HLA* imputation was validated using the sequenced cohort with an overall allele accuracy of >99.9%.

### 10x sequencing of peripheral blood mononuclear cells

PBMCs were obtained from 16 French patients with anti-NMDAR encephalitis and 16 French matched controls using Ficoll isolation and stored in liquid nitrogen until use. Median age at disease onset for cases was 24 years (range 3–48) and 12 (75%) were female. Four (25%) had ovarian teratomas and the remaining ones were non-paraneoplastic. Median delay between disease onset and PBMC collection was 44 days (range 8–1220); 4 patients were untreated at sample collection, 6 received immunotherapy close to the blood drawn (< 3 weeks, “short-term treatment”), and 6 were treated more than 3 weeks before sample collection (“long-term treatment”; see **Supplementary Table 3**).

Single cell libraries were prepared using 10x as instructed by the manufacturer. Briefly, individual PMBCs were thawed and washed with complete RPMI medium (RPMI (Cat# 61870–036, Gibco) supplemented with 10% fetal bovine serum (FBS) and 1% penicillin/streptomycin) and then counted. An equal number of live PBMCs from each sample was pooled and loaded into a 10x chip. Single cell 3’ and 5’ libraries were prepared by Chromium Single Cell 3’ Reagent Kits (v3.1 Chemistry) and Chromium Next GEM Single Cell V(D)J Reagent Kits v1.1 with Feature Barcode technology for Cell Surface Protein, respectively. These libraries were next sequenced on a HiSeq4000 platform at Stanford Genomics for a paired end 2x150 run with a depth of >20,000 read pairs per cell. Sequencing data were processed using the 10x Genomix Cell Ranger v6.0. Single cell was identified using demuxlet (47).

### Phenotype clustering

**Figure 1:**
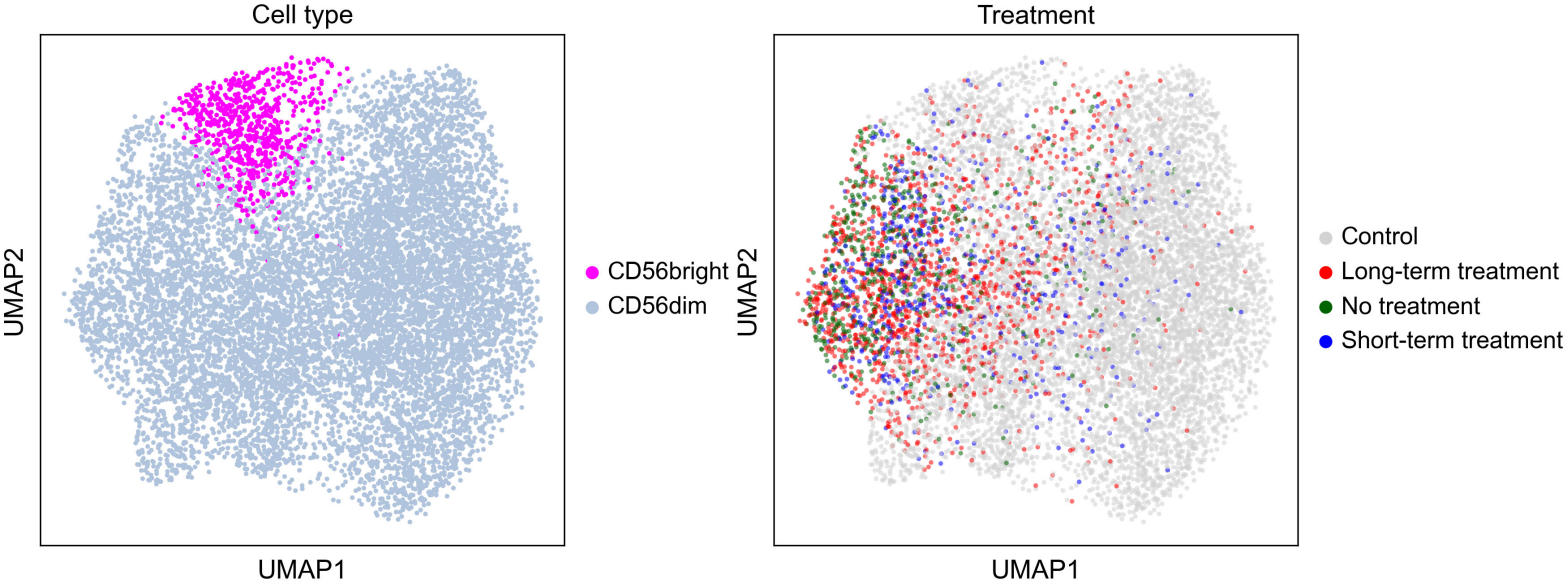
Uniform Manifold Approximation and Projection by cell type and treatment.

10x sequencing results of single cell 3’ and 5’ libraries were imported into Python, concatenated into a single dataset, and analyzed using Scanpy standard workflow (48). Briefly, cells with less than 200 genes were removed from the dataset. Genes that were found in less than 4 cells or encoding hemoglobin/immunoglobulin sequences were removed. Total counts per cell were normalized. Each cell was assigned to a respective stage of cell cycle using a publicly available list of cell cycle genes (49). Next, total counts, cell cycle and mitochondrial content were regressed out. Highly variable genes were identified and used to perform PCA. The first 20 PC were used as an input for Shared Nearest Neighbor clustering and for embedding using the Uniform Manifold Approximation and Projection (UMAP) (**Figure 1**). Marker genes for each cluster were identified computationally using default Scanpy settings. The clusters were manually annotated according to their gene expression pattern. To further identify and cluster NK cells, single cells of PBMCs expressing high level of *NKG7* and *GNLY* were extracted and re-clustered as above, from which NK cells that were *NKG7/GNLY/CD7* positive and *CD3/CD14/CD19* negative were extracted and re-clustered (50–53).

### FACS sorting

NK cells were sorted as previously described (54). Briefly, PBMCs were recovered in complete RPMI medium overnight at 37°C, 5% CO2. Cells were stained with the following combination of antibodies: Brilliant Violet 421^®^ (BV421) (Pacific Blue)-CD7 (clone, CD7–6B7, Cat# 343132), Alexa Fluor^®^ 488 (AF488)-CD14 (clone, HCD14, Cat# 325610), AF700-CD16 (clone, B73.1, Cat# 360718), BV605-CD57 (clone, HNK-1, Cat# 393304), APC-CD3 (clone, UCHT1, Cat# 300412), BV650-CD19 (clone, HIB19, Cat# 302238), APC/Cy7-CD20 (clone, 2H7, Cat# 302314), and PE-CD56 (clone, QA17A16, Cat# 985902). Cells of CD7^+^CD3^-^CD14^-^CD19^-^ were sorted in bulk with BD ARIA II at Stanford shared FACS facility (SSFF). Propidium iodide (PI) was added to separate live cells. Data were analyzed using FlowJo (v10.0.8r1). Sorted cells were immediately loaded into a 10x chip and phenotype analysis were conducted as above.

### Standard protocol approvals, registrations, and patient consents

This study was approved by local ethics committees (IGNITE, #65073), and written informed consent was obtained from all the patients for the storage and use of biological samples and clinical information for research purposes.

### Statistical analysis

#### *KIR* and *HLA* allele association

To avoid stratification issues due to different case-control matching ratios in each ethnic group, a logistic regression controlling for the three first PC (ethnicity) was conducted for each ethnic group and a meta-regression on each allele’s effects was performed using METAL (55). For clarity, we report *HLA* and *KIR* associations with anti-NMDAR encephalitis in all cases and controls in one set of analyses, and in European descent (the largest group) only in another.

*HLA* allele analysis was explored as previously conducted (21); multiple testing was controlled by comparing alleles having a carrier frequency of at least 5% in any group (cases or control) and by using Bonferroni correction. For non-framework *KIR* genes, we first studied if cases versus matched controls differed in presence/absence of each *KIR* genes and in the mean number of each specific *KIR* genes. Following this, we studied allelic associations comparing % individuals with each allele in cases versus matched controls (carrier frequency differences). In cases where a positive association was found (specific *KIR2DL4* and *KIR3DL3* alleles, presence of *KIR2DL5B*), we next analyzed if associations were independent of each other (versus the result of linkage). To do so, we iteratively controlled for the most significant alleles/presence of genes until no significant association remained. Associations were also conducted in teratoma vs. non-teratoma cases, and in teratoma plus non-teratoma vs. controls.

Following these analyses, for non-framework genes, we next explored if cases versus matched-controls differed in effective pairs of *KIR-HLA* ligand. Finally, as these receptors can be either inhibitory or activating (24), we compared the amount of inhibitory and activating inputs NK cells were likely to receive in cases versus controls.

#### Combined HLA-KIR association studies

HLA-KIR interaction associations in anti-NMDAR patients vs. controls were also modeled (30, 32). To do so, we listed all possible KIR interactions with HLA (categorized as A3, A11, Bw4, C1, C2) and assigned them a negative or a positive value from 1–3 depending of each pair effect based on the existing literature for each HLA-KIR pair (24, 25, 27, 30, 31). We then computed and compared the resulting scores in cases and controls using generalized linear and ordinal regression after control of first three PC.

#### Single cell analysis

For single cell RNA-sequencing studies, a linear regression was fitted to study differences in frequency between CD56^dim^ and CD56^bright^ and in gene expression between cases and controls, and controls and each stage of treatment using an ordinal categorization (in order: control, long-term treatment, short-term treatment, no treatment). Similarly, cells with a T lymphocyte (CD3^+^) and NK cell (CD3^-^CD56^+^) were separated and % population carrying *KIR* genes of interest compared between controls and patients as described above. Differences in other gene expression between cells positive for these *KIRs* were also compared in disease vs control. Population age, sex, and PC were controlled for but removed from the model due a lack of significance.

#### Statistical significance

We report FDR (false discovery rate) corrected p-values in alleles with >5% in either case or control carrier frequency for all analyses. Uncorrected p-values are also reported in exploratory analyses and when sample sizes are small, as indicated in each table. Analyses were conducted in R 4.1.0 (R Core Team, 2021) (56), using a significance level of p values < 0.05.

## Results

### Weak *HLA class II* association with DRB1*01:01~DQA1*01:01~DQB1*05:01 in anti-NMDAR encephalitis

Using the largest sample possible (imputed *HLA*), controlling for ethnicity/PC (within each ethnic group) and conducting a meta-analysis, a complex *HLA* association pattern was revealed, with multiple weakly significant *HLA class II* allele associations. These included an increase in frequency of DRB1*01:01~DQA1*01:01~DQB1*05:01, and DRB1*11:01, and a protective effect of DRB3*03:01 (**Table 1**). The DRB1*01:01~DQA1*01:01~DQB1*05:01 increase was more significant in non-teratoma cases (**Supplementary Table 4**). This finding contrast with recent works have outlined that when autoimmune encephalitis is paraneoplastic, *HLA* associations are frequently weaker or non-existent (19, 57). Further, DRB1*01:01~DQA1*01:01~ DQB1*05:01 was also most significant in the sequenced European descent population, doubling the risk of anti-NMDAR encephalitis (**Supplementary Tables 5**, **6**). Finally, we specifically analyzed the carrier frequencies of DQB1*05:02, A*11:01 and A*02:07, as these have recently been reported in a large Chinese cohort (23); we confirm the association with the former but not with the class I alleles (**Supplementary Table 7**).

**Table 1:** Meta-regression of HLA association effects in imputed anti-NMDAR cases vs. controls analyzed using a Generalized Linear Equation modeling after control of first three intra-ethnic Principal Components.

Allele	Estimation	Standard Error	Min	Max	Cases (n=412)*	Controls (n=2784)*	Effect	Std. Error	Estimated OR	P-value**	Direction***	Heterozygosity P-value
DRB3*03:01	5.107	4.610	2.353	17.647	4.497 (21)	9.355 (251)	-0.973	0.280	0.38	2.05E-03	——	0.7340
DRB1*11:01	22.949	3.394	18.182	28.916	21.888 (102)	15.692 (416)	0.471	0.139	1.60	3.05E-02	++++++	0.7291
DQA1*01:01	26.508	8.044	15.294	37.647	23.431 (112)	18.599 (494)	0.415	0.137	1.51	3.88E-02	+++-+-	0.0011
DRB1*13:02	4.457	4.249	1.205	18.182	4.292 (20)	8.714 (231)	-0.935	0.307	0.39	4.79E-02	——	0.8476
DRB1*01:01	22.512	7.057	0.000	32.530	18.670 (87)	15.504 (411)	0.450	0.153	1.57	4.79E-02	-++-+-	0.0004
DQB1*05:01	29.377	9.378	15.385	42.353	26.543 (129)	21.632 (599)	0.374	0.1305	1.45	7.47E-02	+++-+-	0.0040

*Cases and controls with imputation probability < 0.3 were excluded from the meta-analysis.

**FDR corrected.

***Cohort order: African in Affymetrix, East Asian in Affymetrix, European in Affymetrix, European in Illumina, European in PMRA, European in PMRA (replication), non-European in PMRA (replication); “+”: increased in cases, “-”: decreased in cases.

### Copy number variation analysis effects suggest increased presence of *KIR2DL5B* in anti-NMDAR encephalitis

Most *KIR* genes display copy number variations (CNVs). Using a meta-analysis of all cohorts we found that the number of *KIR2DL5B* copies was higher in cases versus controls overall (**Table 2**; **Supplementary Table 8**). This was significant exclusively in European descent cases (**Supplementary Tables 9**, **10**); only in the very small cohort of Asian American was the trend direction reversed (**Table 2**; **Supplementary Table 8**). Of note, as *KIR2DL5* was largely represented by KIR2DL5B*00201, only presence of *KIR2DL5B* was significantly increased (**Supplementary Table 11**).

**Table 2:** Meta-regression of Additive Count Number Variation effects in all cases and controls, analyzed using General Linear Regression modelling after control of first three intra-ethnic Principal Components.

Allele	Effect	Std. Error	P-value	P-value FDR corrected	Direction*	Heterogeneity P-value
KIR2DL3	-0.055	0.040	0.1743	0.3835	—+	0.9663
KIR2DS3T	0.022	0.017	0.2016	0.4032	?+-++	0.7370
KIR2DL1	0.024	0.033	0.4665	0.6842	++–+	0.8283
KIR2DL2	0.055	0.040	0.1719	0.3835	++++-	0.9571
KIR2DS4Del	-0.002	0.044	0.9714	0.9714	+–+-	0.8354
KIR2DL5	0.037	0.050	0.4628	0.6842	++—	0.7386
KIR3DL2	N/A	N/A	N/A	N/A	N/A	N/A
KIR2DP1	0.009	0.033	0.7786	0.9714	-+–+	0.7344
**KIR2DL5B**	**0.101**	**0.028**	**0.0003**	**0.0056**	**+++–**	**0.1845**
KIR2DL4	0.041	0.016	0.0108	0.1191	-++++	0.2680
KIR3DL3	N/A	N/A	N/A	N/A	N/A	N/A
KIR2DS3	0.054	0.037	0.1459	0.3835	++—	0.7279
KIR2DS3C	0.044	0.031	0.1511	0.3835	+++–	0.8106
KIR2DS5	-0.006	0.033	0.8523	0.9714	+—	0.9992
KIR2DS1	-0.041	0.034	0.2214	0.4059	+—	0.5479
KIR3DS1	0.003	0.030	0.9148	0.9714	+—+	0.7697
KIR3DL1	0.056	0.036	0.1182	0.3835	+++++	0.9586
KIR2DS4wt	0.055	0.039	0.1558	0.3835	-++-+	0.5115
KIR2DS2	0.058	0.040	0.1439	0.3835	++++-	0.8043
KIR2DS5C	0.011	0.007	0.1234	0.3835	-+—	0.6663
KIR2DS5T	0.005	0.028	0.8687	0.9714	+-+-+	0.7893
KIR2DL5A	-0.031	0.034	0.3713	0.6284	+—+	0.5406

N/A, not applicable.

*Order of cohorts: African, European, Asian, African-European, Asian-European; “+”, increased in cases; “-”, decreased in cases; “?”, not examined in cohort and thus has a smaller corresponding weight.

Bold values means significant.

### KIR2DL4*00103 and KIR3DL3*00302 associations in anti-NMDAR encephalitis

Comparison of alleles across other loci was first conducted only considering alleles that have amino acid changes, with FDR correction for each locus. As this revealed no significant differences, we explored all known alleles and this revealed three other independent FDR-corrected significant findings (**Table 3**), two of which were present in the European descent only cohort (**Supplementary Table 12**). The same findings were found using a meta-analysis (**Supplementary Table 13**), and the finding did not differ in patients with teratoma versus non-teratoma cases (**Supplementary Table 14**).

**Table 3:** KIR association effects in all anti-NMDAR cases versus controls, analyzed using a Generalized Linear Equation modeling after control of three Principal Components and iteratively controlling for significant alleles.

Iteration	Allele	OR	Cases (n=323)	Controls (n=1519)	P-value		
					Allele*	KIR2DL4*00103	KIR3DL3*00302
1	KIR2DL4*00103	1.98	0.254 (82)	0.125 (190)	6.60E-04	N/A	N/A
2	KIR3DL3*00302	4.44	0.053 (17)	0.013 (20)	4.61E-04	2.09E-05	N/A
3	KIR2DL1*00401	1.60	0.272 (88)	0.185 (281)	2.35E-02	1.09E-05	1.38E-04

N/A, not applicable.

*P-value corrected for multiple comparisons.

One association signal was due to increased telomeric framework gene allele KIR2DL4*00103 in patients with anti-NMDAR encephalitis overall (25.4% vs. 12.5%, OR=1.98, p=6.60×10^-4^, **Table 3**). In addition, presence of centromeric framework gene KIR3DL3*00302, a rare allele only carried by 1.3% of European descent controls was also increased to 5.3% (OR=4.44, p=4.61×10^-4^, **Table 3**). As mentioned, the two effects were independent of each other and carrying KIR2DL4*00103 was independently associated, not surprisingly since they are located on two unlinked segments. Finally, a weak effect of KIR2DL1*00401 remained (OR=1.6, p=2.35×10^-2^), which as mentioned above was not significant in European descent alone and is thus not discussed further (**Table 3**).

As *KIR3DL3* and *KIR2DL5B* are both centromeric, and KIR3DL3*00302 is rare, we conditioned the KIR3DL3*00302 association by presence of *KIR2DL5B* and found these effects to be largely independent (**Supplementary Table 15**). The KIR2DL4*00103, *KIR2DL5B*, KIR3DL3*00302 effects were significantly increased in patients with teratoma and non-teratoma cases with a larger effect in the former group (**Supplementary Table 14**). Similar results were obtained using a meta-analysis per ethnic groups (**Supplementary Table 13**). Meta-regression analysis also identified another rare allele, KIR3DL2*10701, associated with the disease (OR=5.47, p=0.007) and which was removed from the Caucasian analysis due to low carrier frequency. KIR2DL1*00401 was associated with a higher risk of developing the disease in the whole cohort but was not confirmed in the meta-regression.

### Balance of KIR activation/inhibition and HLA ligand pairs

We next explored if specific KIR-HLA ligand interactions (presence of ligand and receptor in the same individual) differed between European descent cases and controls using generalized linear regression after control of first three PC (**Supplementary Tables 16**, **17**). This was also done by meta-regression across all ethnic groups (**Supplementary Tables 18**, **19**) using approximate scores reflecting strength of signaling as reported in **Supplementary data**. As can be seen, KIR2DL1_C2 and KIR2DL2_C1_C2 inhibitory scores were significantly higher in cases versus controls. As a final analysis, **Supplementary Table 20**, a total inhibitory score, total excitatory score, and a sum of both (excitatory minus inhibitory) was compared using a formulation described in **Supplementary data**, revealing no significant difference. It is noteworthy that none of the KIR we identified in the association analysis bind to classical HLA class I ligands.

### *HLA-G* genotyping

Since HLA-G is the ligand of KIR2DL4 (58), we also analyzed differences in *HLA-G* genotypes between cases and controls. However, we found no differences in either *HLA-G* 14-bp Insertion/Deletion 3’UTR promotor polymorphism (**Supplementary Table 21**) or in the frequency of HLA-G*01:05, the rare null allele (**Supplementary Table 22**).

### Differences in NK cell frequencies and gene expression

To assess the immune cell type most related to KIRs, CD56^dim^ or CD56^bright^ cells were next sorted and examined for gene expression (**Table 4**). This was then used to cluster NK cells into CD56^dim^ or CD56^bright^ subtypes across cases by phase of therapy and controls (**Table 4**; **Supplementary Table 21**). Using this method, percentage of CD56^dim^ or CD56^bright^ NK cells did not differ between cases and controls nor across stages of therapies (coded as controls, long-term treatment, short-term treatment, no treatment), although a non-significant decrease in NK CD56^bright^ cells was found in untreated cases vs. controls.

**Table 4:** Gene expression analysis between CD56^bright^ and CD56^dim^ NK cells using Generalized Linear Models and controlling for Age and Sex.

Gene	CD56^bright^ vs. CD56^dim^					Cases vs. Controls						
	Intercept	Beta	P-value*	CD56bright	CD56dim	Intercept	Beta	P-value*	Controls	Long-term treatment	Short-term treatment	No treatment
NKG7	38972	-08639	134E-09	3.166 ± 0.504	4.028 ± 0.313	4009	-0196	153E-04	4.135 ± 0.221	3.903 ± 0.080	3.713 ± 0.286	3.577 ± 0.325
NCAM1 (CD56)	01240	02777	421E-09	0.423 ± 0.195	0.145 ± 0.064	0178	-0033	153E-02	0.206 ± 0.063	0.160 ± 0.063	0.161 ± 0.085	0.113 ± 0.075
FCGR3A (CD16)	06225	-04943	172E-07	0.085 ± 0.143	0.580 ± 0.386	0691	-0199	248E-03	0.751 ± 0.365	0.388 ± 0.181	0.265 ± 0.182	0.270 ± 0.119
KLRC1 (KLRC1)	02479	05604	111E-05	0.906 ± 0.542	0.344 ± 0.218	0512	-0103	218E-02	0.495 ± 0.245	0.319 ± 0.131	0.364 ± 0.232	0.172 ± 0.131
NCR3 (CD337)	04770	-02598	489E-05	0.233 ± 0.190	0.492 ± 0.229	0495	-0081	481E-02	0.534 ± 0.145	0.383 ± 0.142	0.499 ± 0.378	0.276 ± 0.127
KIR2DL1	01049	-00689	534E-05	0.026 ± 0.053	0.095 ± 0.064	0123	-0012	261E-01	0.097 ± 0.051	0.073 ± 0.041	0.085 ± 0.079	0.084 ± 0.095
KLRC3 (NKG2E)	01552	02564	534E-05	0.453 ± 0.271	0.195 ± 0.125	0238	-0025	369E-01	0.239 ± 0.119	0.200 ± 0.158	0.211 ± 0.174	0.164 ± 0.078
B3GAT1 (CD57)	00134	-00165	124E-04	0.000 ± 0.000	0.016 ± 0.020	0015	-0007	438E-02	0.024 ± 0.022	0.009 ± 0.008	0.005 ± 0.011	0.006 ± 0.008
KIR2DL3	00814	-00531	151E-03	0.027 ± 0.057	0.081 ± 0.056	0086	-0011	302E-01	0.082 ± 0.054	0.065 ± 0.028	0.083 ± 0.056	0.042 ± 0.043
KIR3DL2	01160	-00590	151E-03	0.033 ± 0.066	0.092 ± 0.064	0098	0010	398E-01	0.081 ± 0.062	0.084 ± 0.061	0.095 ± 0.076	0.098 ± 0.050
KLRC2	02395	02400	151E-03	0.453 ± 0.328	0.213 ± 0.160	0302	-0029	393E-01	0.277 ± 0.187	0.181 ± 0.121	0.205 ± 0.138	0.239 ± 0.239
KIR2DL4	00953	00955	206E-03	0.133 ± 0.156	0.038 ± 0.024	0085	-0005	398E-01	0.055 ± 0.044	0.050 ± 0.022	0.039 ± 0.016	0.055 ± 0.052
GNLY	45563	03235	992E-03	4.735 ± 0.366	4.415 ± 0.530	4740	-0033	713E-01	4.546 ± 0.402	4.389 ± 0.447	4.195 ± 0.769	4.534 ± 0.304
KIR3DL1	00595	-00631	992E-03	0.029 ± 0.109	0.091 ± 0.079	0075	-0019	141E-01	0.089 ± 0.080	0.095 ± 0.059	0.085 ± 0.103	0.049 ± 0.048
KLRG1	01179	-00761	992E-03	0.086 ± 0.110	0.161 ± 0.099	0150	-0035	483E-02	0.190 ± 0.108	0.132 ± 0.070	0.128 ± 0.069	0.077 ± 0.062
KLRB1 (CD161)	20964	-02592	471E-02	1.981 ± 0.616	2.239 ± 0.297	2168	-0139	614E-03	2.331 ± 0.176	2.235 ± 0.144	2.067 ± 0.388	1.873 ± 0.363
KLRK1 (NKG2D)	06562	01177	259E-01	0.791 ± 0.393	0.674 ± 0.289	0912	-0208	225E-05	0.910 ± 0.178	0.519 ± 0.154	0.487 ± 0.256	0.342 ± 0.082
NCR1	01156	-00221	549E-01	0.114 ± 0.135	0.136 ± 0.073	0147	-0030	382E-02	0.154 ± 0.059	0.137 ± 0.054	0.131 ± 0.109	0.053 ± 0.048
CD7	19797	-00354	836E-01	1.906 ± 0.457	1.941 ± 0.249	1979	0041	393E-01	1.881 ± 0.211	1.975 ± 0.221	2.018 ± 0.393	2.048 ± 0.161
KLRC4 (NKG2F)	01007	00054	847E-01	0.096 ± 0.110	0.091 ± 0.062	0113	-0033	153E-03	0.127 ± 0.037	0.047 ± 0.024	0.088 ± 0.083	0.032 ± 0.028
KLRD1 (CD94)	13727	00430	847E-01	1.378 ± 0.576	1.336 ± 0.460	1700	-0318	474E-05	1.648 ± 0.335	1.219 ± 0.130	1.113 ± 0.426	0.671 ± 0.304
KLRF1	07242	-00258	847E-01	0.888 ± 0.363	0.910 ± 0.384	0922	-0176	122E-02	1.092 ± 0.384	0.754 ± 0.245	0.815 ± 0.304	0.582 ± 0.225
KIR3DL3	00007	431E-05	966E-01	0.001 ± 0.005	0.001 ± 0.003	0002	0000	734E-01	0.000 ± 0.001	0.003 ± 0.006	0.000 ± 0.000	0.000 ± 0.000

*P-values FDR corrected for multiple comparisons. Covariates not shown due to lack of significance. Mean of means shown.

In term of gene expression profile, as expected, CD56^bright^ mostly differed from CD56^dim^ as follows: increased *CD56/NCAM1* (neural cell adhesion molecule 1), *KLRC3/NKG2E* (killer cell lectin like receptor C3) and *KLRC1/NKG2A* plus decreased *NKG7* (natural killer cell granule protein 7), *CD16/FCGR3A* (Fc gamma receptor IIIa), *KIR2DL1* and *CD57/B3GAT1* (beta-1,3-Glucuronyltransferase 1). *KIR2DL4* was also significantly increased in CD56^bright^ as expected.

We next compared gene expression within NK cells in cases versus controls. Because only four untreated cases were available (in almost all cases, anti-NMDAR encephalitis cases receive immunosuppression early in the disease course), to test a difference by disease status, we expected a gene to be most different in these four cases and to recover closer to control values as treatment progressed and the case resolved. For this reason, an ordinal model (controls, long-term treatment, short-term treatment, no treatment) was set up looking at expression, also controlling for age and sex; however, the results did not change (data not shown).

In general, we found that expression of many genes was decreased in anti-NMDAR encephalitis vs. controls and by phase of therapy, most notably *KLRD1/CD94*, *KLRK1/NKG2D*, *NKG7*, *NCAM1/CD56*, *KLRB1/CD161* and *KLRC4/NKG2F*. Importantly, *KIR2DL4* did not differ. *KIR3DL3* expression was also examined but it was low (data not shown) and based on its recently reported cellular distribution in γδ and CD8^+^ T cells (59) that was not clearly observed in our dataset (data not shown), data for this gene was not reported. *KIR2DL5B* expression was also not observed, in line with reports that it is generally silenced (60–62).

## Discussion

In this work, we conducted *HLA* imputation/sequencing and *KIR* sequencing in the largest reported genetic analysis of anti-NMDAR encephalitis cases, using a recently described pipeline. Anti-NMDAR encephalitis was a particularly interesting candidate disease in which to explore effects of KIR and HLA, given it is autoimmune and associated with both viral infection (post-HSV encephalitis) and tumors.

We found a weak *HLA* association with DRB1*01:01~DQA1*01:01~DQB1*05:01, a frequent haplotype, that will need to be replicated. Previously, weak associations were reported with B*07:02 in White adult patients (13), DRB1*16:02 in Chinese populations (14), and DRB1*15:02 in a pediatric Thai cohort (63). More recently, a large Chinese study revealed rather strong associations with DQB1*05:02, A*11:01 and A*02:07 (23). Altogether, these results suggest that, in contrast to other autoimmune encephalitides with strong and homogeneous HLA associations across different ethnicities, such as those related to antibodies against leucine-rich glioma-inactivated 1 (LGI1) or IgLON5 (21, 64), the HLA associations in anti-NMDAR encephalitis are more diverse and likely reflect a less significant role in the pathogenesis of the disease.

More interestingly, we found an association with increased number of copies of KIR2DL5B and two allele associations in framework genes, KIR2DL4*00103 and KIR3DL3*00302. *KIR2DL4* is a framework gene. KIR2DL4 is likely an activating receptor that recognizes HLA-G as its ligand (58). HLA-G is highly expressed in trophoblast, although expression is also present in thymus, cornea, and pancreas islet (65, 66). HLA-G is also present as a secreted protein. The KIR2DL4-HLA-G interaction may be particularly important in the context of the feto-maternal interface. Indeed, KIR2DL4 is primarily expressed in CD56^bright^ NK cells (as confirmed in this study), cells that also include decidual NK cells. Interestingly, KIR2DL4 is mostly intracellular and only a small portion is expressed at the surface of NK cells (58). In this context, CD56^bright^ NK cells, a subtype of NK cells considered less differentiated and more cytokine-producing than cytotoxic, is likely useful to induce tolerance to trophoblastic invasion though KIR2DL4-HLA-G interactions. A similar role may be played in the context of virally infected or cancerous cells. Ectopic expression of HLA-G in tumor and in virally infected cells has also been reported, where it binds leukocyte immunoglobulin-like receptors (LILR) subfamily B and likely operates as a checkpoint inhibitor, dampening NK cell activity (66). KIR2DL4*00103 amino-acid sequence does not differ from the most common KIR2DL4*00102, an allele that was not associated with anti-NMDAR encephalitis in our dataset (31.2% vs. 33.6% in controls, ns, data not shown). Thus, the difference observed here with KIR2DL4*00103, if replicated, is more likely to result from differences in expression.

To complement this finding, we also explored whether *HLA-G* genotypes differ across cases and controls, finding no differences in either *HLA-G* promotor polymorphisms or in the frequency of the rare *HLA-G* null allele across cases and controls (**Supplementary Tables 19**, **20**). This, together with the fact KIR2DL4*00103 does not differ in sequence with KIR2DL4*00102 makes this pathway unlikely to be strongly involved in anti-NMDAR encephalitis. Sequencing of additional samples will be needed to confirm the association with non-coding allele KIR2DL4*001.

Another finding was an association with the rare KIR3DL3*00302 allele. Similar to the above, KIR3DL3*00302 does not differ in amino acid sequence from the more common KIR3DL3*00301, which did not differ in our data set (22.9 vs. 25.0, ns, data not shown). KIR3DL3 ligand is HHLA2 (Human endogenous retrovirus-H long terminal repeat-associating 2), a variant of the B7 family that is mostly expressed in monocytes, but is also present in trophoblastic cells. Like HLA-G, it may function as a checkpoint inhibitor. Interestingly, KIR3DL3 was recently shown to be mostly expressed in CD8^+^ T cells and γδ T cells (59), a finding we could not confirm in our single cell sequencing due to low expression of this receptor in peripheral blood. As for the *KIR2DL4* association, the fact the allele does not differ from a more common variant that is not associated is not strongly supportive of involvement, although the fact both KIR2DL4 and KIR3DL3 have related function is intriguing.

A more solid finding pertained to increased copy number variation of *KIR2DL5B*. *KIR2DL5* is present as two extremely similar genes, *KIR2DL5A* (telomeric) and *KIR2DL5B* (centromeric). KIR2DL5 was considered an orphan molecule until recent studies identified it as a new binding partner of poliovirus receptor (PVR, also known as CD155) using high-throughput screening of receptor-ligand interactions (67–69). KIR2DL5A frequency, a receptor expressed in both innate (NK and γδ T cells) and adaptive (CD8^+^ T cells) immune cells from human peripheral blood (70), did not differ globally in our study. While most *KIR5DL5B* alleles are considered transcriptionally silent because of an impaired RUNX binding site conserved in the promoter region of most KIRs (-97A), most known *KIR2DL5A* alleles and a few *KIR2DL5B* alleles have intact RUNX binding sites and are expressed (70).

Expression of PVR is low or absent in most healthy tissues; however, it is overexpressed on numerous types of tumors, including colorectal cancer, glioma, myeloid leukemia, ovarian cancer, lung cancer, pancreatic cancer, melanoma, and other tumors (71). Accumulating evidence suggests that PVR overexpression induces the immune escape of tumor cells and is associated with a poor prognosis and enhanced tumor progression (70). Besides its tumor-intrinsic roles, PVR participates in multiple immunoregulatory events through finely tuned interaction with the stimulatory receptor DNAX accessory molecule 1 (DNAM-1, also known as CD226) and the inhibitory receptors T cell immunoreceptor with Ig and ITIM domains (TIGIT) and CD96 (72). Further, the KIR2DL5-PVR pathway has been shown to be important in modulating responses of HIV infected cells by NK cells (73). Finally, *KIR2DL5* polymorphisms have been associated with multiple sclerosis, a disease associated with EBV infection (74).

In our study, increased *KIR2DL5B* copy number was found in anti-NMDAR encephalitis (**Table 1**). As expected, the increase was mostly due to KIR2DL5B*00201 (27.5 vs. 19.5%, p=0.03), the most frequent allele, although other alleles, which are rare, were also increased. Interestingly, KIR2DL5B*00201 and most other alleles are characterized by the presence of 162Asp and 174Gly in the D2 domain (exon-5) of the KIR molecule, an area known to be essential to PVR binding (70). As such, most *KIR2DL5B* alleles are not only known to be expressed under normal conditions but also unlikely to bind PVR as their ligand. These changes are characteristic of most *KIR2DL5B* alleles and also shared by KIR2DL5A*00501, the only *KIR2DL5A* allele slightly increased in anti-NMDAR encephalitis (10.6% vs. 8.7%, ns). Interestingly, KIR2DL5A*00501 is also transcriptionally silent (60, 70). Although most *KIR2DL5B* alleles including KIR2DL5B*00201 (and KIR2DL5A*00501) are transcriptionally silent in normal conditions in blood (other tissues have not been tested), demethylation of the promotor could restore expression, thus whether increased *KIR2DL5B* and allele differences in *KIR2DL5A* are relevant to anti-NMDAR encephalitis is still possible. Of note, however, all alleles associated with anti-NMDAR encephalitis are not those binding PVR, thus interaction with another yet unknown ligand would have to be involved.

We next explored gene expression differences in NK cells of patients versus controls across therapy. Interestingly, decreased *KLRD1/CD94*, *KLRK1/NKG2D*, *NKG7*, *NCAM1/CD56*, *KLRB1/CD161* and *KLRC4/NKG2D* were found (**Table 4**). Most of the changes were due to changes observed within the NK CD56^dim^ population, were present in untreated patients and improved partially with therapy (data not shown), as predicted from the analysis we designed. CD94, which is decreased, heterodimerizes with NKG2A and C to interact with HLA-E (27), and is either inhibitory or activating. KLRK1/NKG2D is activating and recognizes MIC and RAET1/ULBP families which appear on the surface of stressed, malignant transformed, and infected cells. It is thought to be important in viral and cancer control and viruses/cancer have adapted mechanisms by which to evade NKG2D responses such as with CMV (75). As for intracellular NKG2F, NKG2D does not pair with CD94; its function is largely unknown. Overall, the profile of these cells is consistent with a population of NK cells that may be more difficult to activate, although it is important to realize circulating NK cells are not representative of tissue homing NK cells, which are mostly CD56^bright^ and cytokine producing. Additional studies will be needed to confirm and expend on these findings.

The main limitations of the current study are the relatively low number of non-European and teratoma-related patients included, which hindered the identification of specific HLA and KIR associations in these subsets of patients, as well as the small number of untreated patients from whom PBMCs were used to investigate NK cell frequencies and gene expression.

In conclusion, our study of anti-NMDAR encephalitis revealed minor changes in KIR polymorphism distribution, although increased *KIR2DL5B* copy number was found. No significant differences in cell numbers for all major cell categories were found, although a trend in decreased CD56^bright^ was observed. Many of these changes could reflect past or current viral infection, or be the result of the autoimmune process. Gene expression in NK cells revealed changes that are predicted to result in less easily activatable cells.

## Data availability statement

Publicly available datasets were analyzed in this study. This data can be found here: https://doi.org/10.7910/DVN/W7QAIR and https://doi.org/10.7910/DVN/AOIFIO.

## Ethics statement

The studies involving humans were approved by IGNITE (#65073) Stanford University. The studies were conducted in accordance with the local legislation and institutional requirements. Written informed consent for participation in this study was provided by the participants’ legal guardians/next of kin.

## Author contributions

VS: Conceptualization, Data curation, Formal analysis, Investigation, Methodology, Software, Validation, Writing – original draft, Writing – review & editing. GL: Conceptualization, Data curation, Formal analysis, Investigation, Methodology, Software, Writing – original draft, Writing – review & editing. SM-C: Data curation, Formal analysis, Investigation, Methodology, Writing – original draft, Writing – review & editing. A-LP: Data curation, Writing – review & editing. GP: Data curation, Writing – review & editing. VR: Data curation, Writing – review & editing. MT: Data curation, Investigation, Writing – original draft, Writing – review & editing. CF: Data curation, Investigation, Writing – original draft, Writing – review & editing. FL: Data curation, Investigation, Methodology, Writing – original draft, Writing – review & editing. GK: Data curation, Investigation, Methodology, Writing – original draft, Writing – review & editing. HJ: Data curation, Writing – review & editing. RD: Data curation, Writing – review & editing. SB: Data curation, Investigation, Methodology, Writing – review & editing. SI: Data curation, Investigation, Methodology, Writing – original draft, Writing – review & editing. AB: Data curation, Writing – review & editing. JV: Data curation, Writing – review & editing. MB: Data curation, Writing – review & editing. DR: Data curation, Investigation, Writing – review & editing. T-JK: Data curation, Writing – review & editing. KC: Data curation, Writing – review & editing. S-TL: Data curation, Writing – review & editing. TK: Data curation, Writing – review & editing. NP: Data curation, Formal analysis, Investigation, Methodology, Writing – review & editing. KK: Data curation, Formal analysis, Investigation, Methodology, Writing – review & editing. AM-M: Data curation, Formal analysis, Investigation, Methodology, Writing – review & editing. JH: Conceptualization, Data curation, Formal analysis, Funding acquisition, Investigation, Methodology, Project administration, Resources, Software, Supervision, Validation, Visualization, Writing – original draft, Writing – review & editing. PN: Conceptualization, Data curation, Formal analysis, Funding acquisition, Investigation, Methodology, Project administration, Resources, Software, Supervision, Validation, Visualization, Writing – original draft, Writing – review & editing. EM: Conceptualization, Data curation, Formal analysis, Funding acquisition, Investigation, Methodology, Project administration, Resources, Software, Supervision, Validation, Visualization, Writing – original draft, Writing – review & editing.
